# Nanopatterning Single-Crystalline Metal Electrodes via Ion Erosion: New Structural Motifs for Model Electrocatalysis

**DOI:** 10.1021/acs.jpclett.5c01465

**Published:** 2025-07-15

**Authors:** Pankaj Kumar Samal, Jan Škvára, Matyas Výhonský, Lukáš Fusek, Michal Ronovský, Viktor Johánek, Maximilian Kastenmeier, Yaroslava Lykhach, Jörg Libuda, Olaf Brummel, Josef Mysliveček

**Affiliations:** † Department of Surface and Plasma Science, Faculty of Mathematics and Physics, 37740 Charles University, V Holešovičkách 2, 180 00 Praha 8, Czech Republic; § Interface Research and Catalysis, ECRC, 9171 Friedrich-Alexander-Universität Erlangen-Nürnberg, Egerlandstraße 3, 91058 Erlangen, Germany

## Abstract

Model studies in electrocatalysis provide valuable insights into complex structure–property relationships and rely heavily on experimental techniques that enable the preparation of electrode surfaces with atomic-scale control over morphology. This work investigates the nanopatterning of single-crystalline metal electrodes through ion erosion. On Pt(111), ion erosion produces surfaces with distinct corrugation, characterized by the formation of erosion pits, an equal proportion of (110) and (100) steps, and a high density of kinks. The obtained structural motifs influence the electrochemical behavior of H/OH adsorption/desorption and surface electrooxidation in a unique manner, illustrating that ion-eroded metal surfaces represent a complex but atomically precise platform for exploring the synergistic action of various types of surface defects on the electrode properties. A general applicability of ion erosion is demonstrated by preparing and characterizing ion-eroded surfaces of Ru(0001) and Cu(111), showing that this method can control the density of surface defects in ways that are challenging to achieve through the traditional Clavilier method. Ion erosion enables the imprinting of a variety of surface configurations, ranging from atomically flat to stepped and disordered structures, on a single-crystal substrate, offering new avenues for designing electrodes with tailored reactivity.

## Graphical abstract

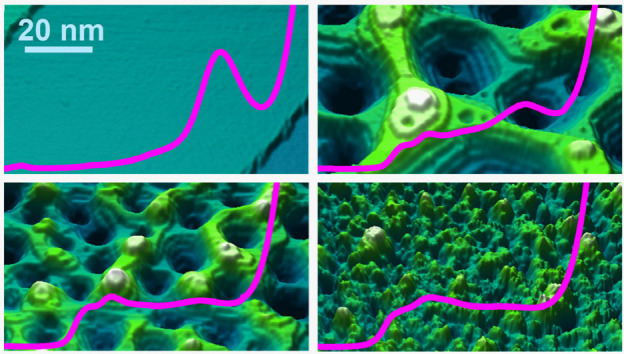

A comprehensive understanding of the electrocatalytic activity of complex nanostructured electrode materials requires model experiments to evaluate the relationships among morphology, chemical composition, and electrochemical properties. These experiments typically involve model catalyst systems with surface structures, compositions, and defect types controlled with atomic-level precision.^1–3^ For metal-based electrocatalysts, the most commonly used model systems are singular and vicinal surfaces of metal single crystals. These surfaces are often prepared using the Clavilier method, where millimeter-sized single crystals are grown from polycrystalline wires, cut and polished to the desired crystallographic orientation, and cleaned via a flame or induction annealing in an appropriate working atmosphere at ambient pressure.^4,5^ The resulting surfaces typically represent ideal terminations of the corresponding bulk structures, with the atomic arrangement and step and kink densities determined by the crystallographic orientation.^6^ Metal single-crystal surfaces remain, to date, the most important systems for understanding structure–property relationships in electrocatalysis.^7–12^ In recent years, there has been a resurgence of surface science-based approaches to model electrode preparation,^13–15^ driven largely by the need to prepare surfaces that are incompatible with conventional ambient pressure annealing, such as reactive metals and metal oxides.^16–19^ Vacuum deposition techniques offer the advantage of functionalizing metal electrodes with materials that are otherwise challenging to obtain via electrochemical deposition.^20,21^

A valuable tool for the controlled nanopatterning of surfaces is ion erosion. By exposing a sample with an appropriate temperature to an ion beam of appropriate energy and orientation, surface atoms can be selectively removed, resulting in self-assembly of atomically well-defined nanostructures and precise control over surface morphology.^22–26^ While ion-eroded single-crystal metal surfaces have been extensively studied in the context of gas-phase catalysis,^27–29^ the present study aims to extend the use of these surfaces to model electrocatalysis.^30,31^ Specifically, we investigate electrochemical properties of ion-eroded Pt(111), Ru(0001), and Cu(111) surfaces and show that ion erosion provides atomic-level control of new structural motifs and structure–property relationships related to the evolving surface corrugation, which are not available on singular or vicinal single-crystalline electrodes.

Our research strategy is based on the model approach that involves the preparation of well-defined nanopatterned single-crystalline metal electrodes under ultrahigh-vacuum (UHV) conditions and transfer of the prepared electrodes to the *ex situ* emersion electrochemical cell under an inert atmosphere of Ar,^32^ (see the Supporting Information for more experimental details). Well-defined nanopatterned Pt surfaces with precise densities of etch pits were prepared by means of ion erosion of Pt(111) single crystals at temperatures between 300 and 700 K, following the approach of ref 33. Examples of scanning tunneling microscopy (STM) images of the as-prepared samples are shown in Figure 1. The complete set of images is provided in Figure S1 and included in the open data set related to this paper.

**Figure 1:**
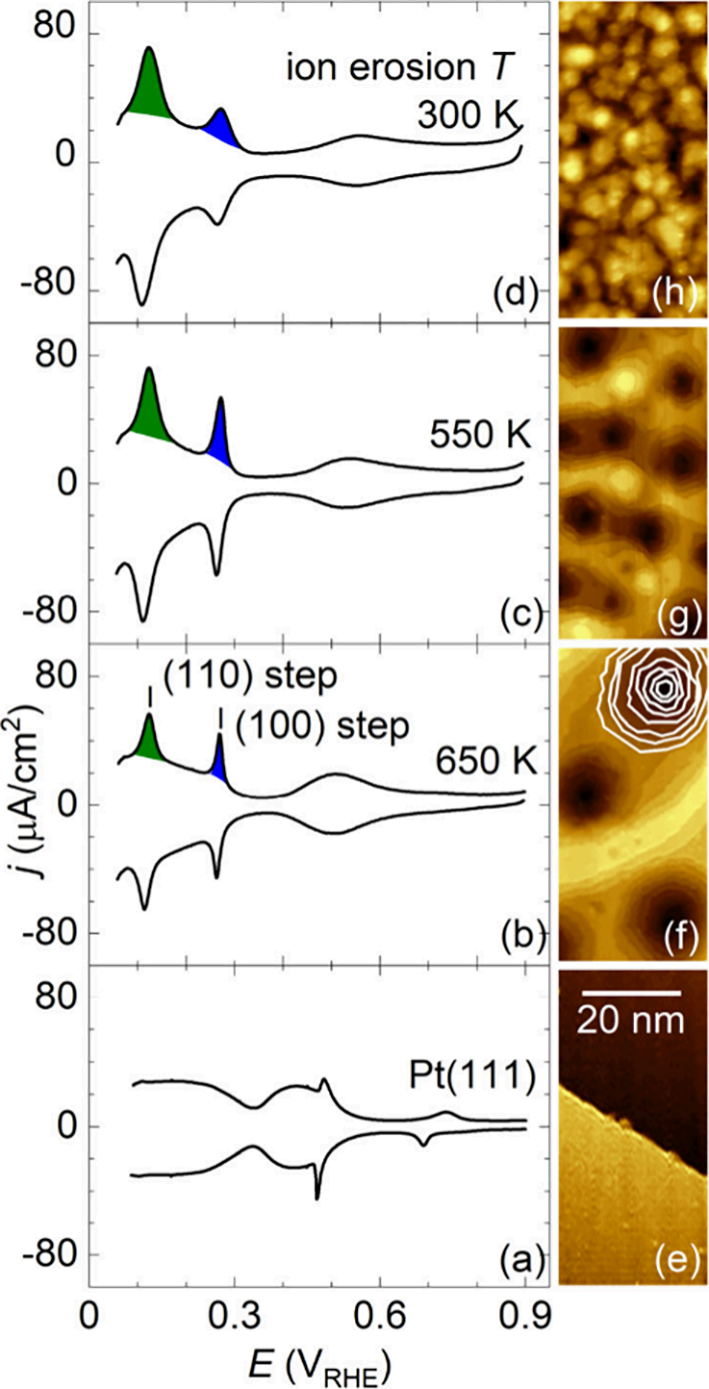
Pt­(111) samples ion-eroded at different temperatures; (a–d) Blank CVs of the ion-eroded samples after transfer from UHV to the EC cell (0.1 M H_2_SO_4_, at 50 mV/s). Marked are CV peaks related to (110) and (100) monatomic steps on Pt(111). Charges in the peaks are highlighted in color. (e–h) STM images of corresponding samples as-prepared and measured in UHV. In panel f, step edges inside an etch pit are outlined.

STM images reveal the formation of nanometer-sized etch pits of a conical shape. Pit density and pit height as a function of the substrate temperature during sputtering correspond to previous observations (panels c and d of Figure S2).^33^ Inside of etch pits, monatomic step edges become apparent for the samples prepared at temperatures of 500 K or higher (panels b and c of Figure 1). Steps are largely equidistant, and the distance between the steps decreases with a decreasing substrate temperature during sputtering (Figure S2e).^33^ Qualitatively, ion erosion of Pt(111) yields surfaces with increased density of monatomic steps. We also prepared a sample ion-eroded at 300 K. On this sample, etch pits and monatomic step edges were not resolved (Figure 1d).

In a separate experiment, samples were prepared under the same conditions and transferred under an inert gas atmosphere to an electrochemical cell where they were characterized in a static electrolyte in a hanging meniscus configuration. Electrochemical treatment of the samples is illustrated in Figure S3. For identification of the electrochemical features related to the surface steps, we performed blank cyclic voltammetry in 0.1 M H_2_SO_4_ at a scan rate of 50 mV/s. Selected cyclic voltammograms (CVs) are listed in Figure 1. The complete set of CVs is provided in Figure S4 of the Supporting Information.

On clean Pt(111), we resolve the hydrogen region between 0.05 and 0.35 V_RHE_ related to H adsorption/desorption and the sulfate adsorption/desorption region between 0.35 and 0.65 V_RHE_.^34^ As the sample preparation temperature decreases and the density of etch pits increases, we observe an increasing intensity of two peaks at 0.11 and 0.26 V_RHE_ in the hydrogen region. These features are attributed to H/OH exchange at monatomic Pt(111) step edges with (110) and (100) orientations, respectively.^35–39^ For ion erosion at temperatures of 500 K and above, the peak at 0.26 V_RHE_, corresponding to the (100) step edges, appears particularly sharp. This indicates the presence of well-separated (100) steps that are largely free of other structural defects.^40,41^ The peak at 0.11 V_RHE_, associated with (110) step edges, shows a comparable amplitude but is broader. When ion erosion is performed at 300 K, the (110) and (100) step edge features become broadened, indicating a less well-defined step structure.

We evaluated the charges in the obtained CVs according to the fitting procedure suggested in ref 42 and plotted the charges associated with (110) and (100) steps in Figure 2. Descriptions of the fitting procedure and fitted curves are shown in Figure S4, and an overview of all charge contributions is shown in Figure S5. We observed an increase of the charge in the (110) and (100) step edge features with a decreasing ion erosion temperature between 700 and 550 K and saturation of the charge between 550 and 300 K. Between 700 and 550 K, the charge in the (110) feature is higher than the charge in the (100) feature by a factor of 1.7 (Figure S6). At 500 and 300 K, the charge in the (110) feature is higher by a factor of 2 (Figure S6).

**Figure 2:**
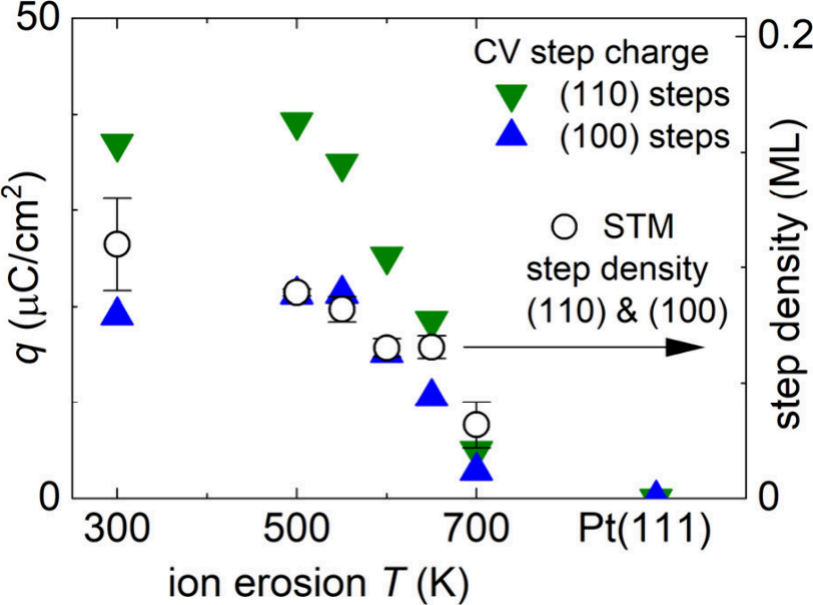
Structure–property relationships in H/OH adsorption/desorption. Left axis (full symbols): charge in the step peaks of the CVs of the ion-eroded Pt(111) samples. Right axis (open symbols): (110) and (100) step densities as determined from STM images. The (110) and (100) step densities are equal. Scales of the step charge (left axis) and step density (right axis) are linked assuming a charge of 1 e^–^ per step site.

To obtain the quantitative correlation between the step charge in CVs and the step density of the ion-eroded samples, we analyzed the STM images of ion-eroded Pt(111) for step density as a function of the ion erosion temperature and plotted the result in Figure 2 (right axis). We note that the step edges on the ion-eroded samples exhibit a distinct curvature, which results in alternating (110) and (100) step segments and an increased density of kink sites. The evaluation procedure thus must account for the densities of (110) step segments, (100) step segments, and kinks separately. The evaluation procedure is illustrated and described in panels a–d of Figure S7. An estimate is also provided for the sample ion-eroded at 300 K, where unresolved step edges are modeled following the approach of ref 41. We can conclude that, for all ion-eroded samples, densities of (110) and (100) step segments in the STM images of the as-prepared samples are equal (panels e and f of Figure S7). This makes ion-eroded Pt(111) samples qualitatively different from Pt(111) samples nanostructured by electrochemical methods, where (110) step edges dominate.^40,41,43^ The maximum step density of ∼0.2 ML is obtained at 300 K (Figure 2 and Figure S7e). The step density decreases with an increasing ion erosion temperature (Figure 2 and Figure S7e). Evaluation of the kink density shows that it is proportional to the step density, accounting for approximately 20% of the step density (Figure S8). Step and kink densities become less well-defined for temperatures of 650 and 700 K due to increasing inhomogeneity of the surface structure (panels e–h of Figure S1).

In a traditional interpretation, both (110) and (100) steps contribute a charge of 1 e^–^ per step site to the CV features due to H adsorption and desorption at steps.^35,36^ This allows correlating the step density determined from STM to an expected step charge, as indicated in Figure 2. We observe that, on ion-eroded Pt(111) samples, STM step density compares favorably to the CV charge of (100) steps. For (110) steps, we observe an excess CV charge by a factor of 1.7 for samples ion-eroded at temperatures between 550 and 700 K and by a factor up to 2 for the samples prepared at 300 and 500 K (cf. Figure S6).

Recent experimental and theoretical results indicate that excess (110) charge in the CVs of stepped Pt surfaces can be a consequence of a mechanism more complex than plain H adsorption/desorption. Koper and Feliu have proven that, on both (110) and (100) step edges, H co-adsorbs with OH.^37–39^ This may increase the charge per step site associated with (110) and (100) step peaks by a contribution from OH desorption accompanying H adsorption.^42^ Co-adsorption of OH was found to be structure-sensitive, being stronger at (110) steps than on (100) steps.^44,45^ Experimental reports of excess CV charge in the (110) step peak yield values between 1 and 1.4.^41,42,45,46^

We propose that the increase in (110) excess charge beyond the factor of 1.4 observed in our experiment can be related to the distinct morphology of the ion-eroded Pt(111) samples, particularly to the presence of kinks at the surface step edges. Reports on H/OH adsorption/desorption at stepped Pt electrodes containing kinks are scarce in the present literature,^47–50^ but they indicate a broadening of the (110) step peak compared to the (100) peak similar to the broadening observed in our experiment (Figure 1b and Figures S3 and S4). Local morphology of kinked steps, eventually in combination with local surface stress release,^10^ is thus expected to further modify the structure-sensitive co-adsorption of H and OH to an extent not previously reported.

We note that conditions of our experiment exclude mechanisms reported to change the distribution of (110) and (100) step edges on the Pt surface, particularly destabilization of (100) step edges. This can be caused by adventitious oxidation of the Pt surface, e.g., by exposure of the hot Pt surface to air^51^ or by electrooxidation at potentials beyond 1.1 V_RHE_ ^40^ and subsequent electroreduction. Potentials for our CV measurements are selected inside the Pt stability range between 0.05 and 0.90 V_RHE_ (panels a–c of Figure S3),^40^ CVs are stable from the first scan (panels a–c of Figure S3), and no peak broadening or splitting indicating the destabilization of (100) steps is observed in the (100) CV peak at 0.26 V_RHE_ (cf. refs 40, 41, and 51 ). In a dedicated experiment, we have evaluated the degree of oxidation of the ion-eroded Pt samples upon the transfer from UHV to the electrochemical (EC) cell (Figure S9).^51^ The degree of oxidation is the same as reported for Pt samples prepared by flame annealing and subsequent cooling in a H_2_/Ar atmosphere, without a destabilization effect on the (100) step population.^51^ Finally, STM images after the emergence of ion-eroded Pt upon CV characterization show no observable changes of the pitted surface morphology (Figure S10). This observation also confirms stability of the kink population on ion-eroded Pt(111) samples.

Structure sensitivity of the hydrogen evolution reaction (HER) and oxygen evolution reaction (OER) on ion-eroded Pt(111) has been tested in single linear sweep voltammetry (LSV) scans into HER and OER regions (panels d and f of Figure S3). The HER on Pt in acid is a fast reaction, and resolving its structure sensitivity requires a dedicated experimental approach.^34^ In our experiment, structure sensitivity of HER is not detected (Figure 3a), and the potential at which the HER current density reaches −300 μA/cm^2^ is constant (Figure 3c). The HER has no influence on the morphology of the investigated samples, as they exhibit the same CVs before and after the HER (cf. panels b and e of Figure S3 and ref 52).

**Figure 3:**
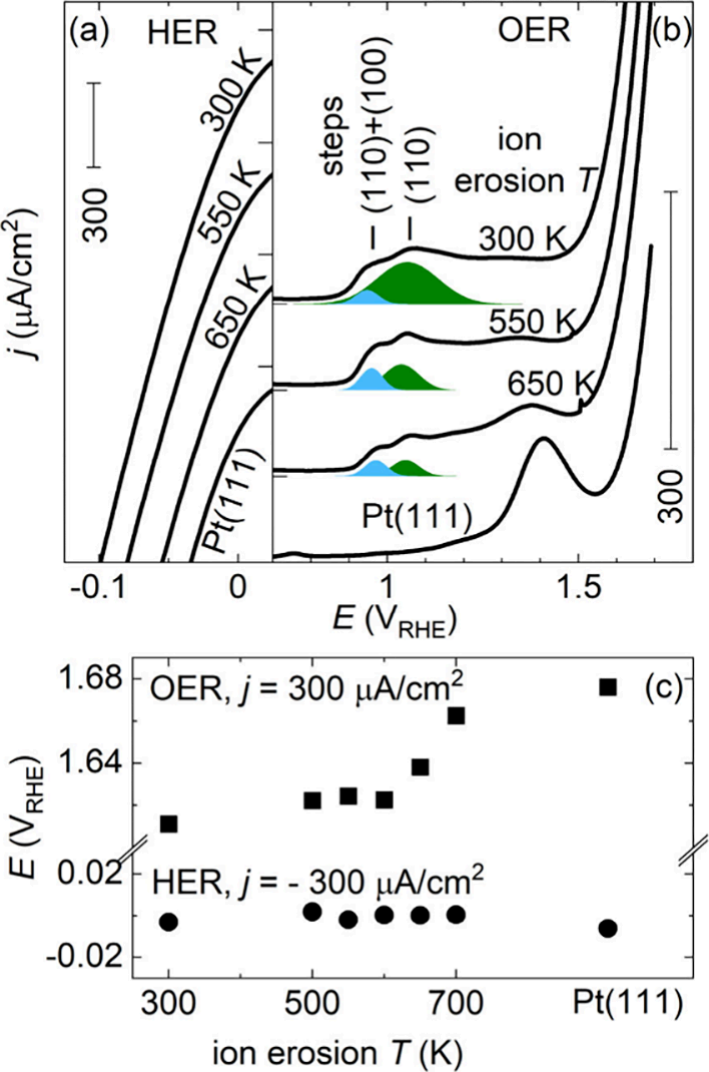
First LSV scans of Pt(111) samples ion-eroded at different temperatures to the (a) HER and (b) OER potential regions (0.1 M H_2_SO_4_, at 50 mV/s). In panel b, peaks assigned to oxidation of (110) and (100) surface steps are marked and peak charges are highlighted in color. (c) Potential at which the absolute value of HER/OER currents reaches 300 μA/cm^2^. Note the axis break on the vertical axis. LSVs in panels a and b are offset by −400 and +100 μA/cm^2^, respectively. Zero points of the offset curves are indicated.

The OER in sulfate-containing electrolytes is strongly influenced by anion adsorption.^40,43^ For Pt(111), sulfate hinders the surface oxidation up to about 1.4 V_RHE_, resulting in a single oxidation peak preceding the OER onset (Figure 3b).^53^ Sulfate ions, however, do not adsorb at steps.^53^ As the step density on our samples increases from a minimum on singular Pt(111) to a maximum on Pt(111) ion-eroded at 300 K, we thus observe evolution of step oxidation peaks at 0.95 and 1.05 V_RHE_ (Figure 3b)^53^ and shift of the OER onset toward lower potentials (Figure 3c).

Experiments on stepped Pt surfaces reported in the literature identify the observed step oxidation peaks with oxidation of (110) and (100) steps as follows: the peak at 0.95 V_RHE_ is observed during oxidation of both (110) and (100) step edges, and the peak at 1.05 V_RHE_ is observed during oxidation of (110) step edges.^46,53,54^ Oxidation charge in the peaks has been reported 1 e^–^ per step edge site for (100) steps (in the peak at 0.95 V_RHE_)^54^ and 2–3 e^–^ per step edge site for (110) steps (1 e^–^ in the peak at 0.95 V_RHE_ and 1–2 e^–^ in the peak at 1.05 V_RHE_).^46,53^

Step oxidation charges on ion-eroded Pt(111) can be determined by fitting the OER LSVs from Figure 3b for step and terrace contributions according to refs 46 and 53 . The obtained step oxidation charges are shown in Figure 4, and fit details and an overview of all oxidation charges are shown in Figures S11 and S12, respectively. To compare step oxidation charges with step densities of ion-eroded Pt(111) samples, we plot the (110) and (100) step densities obtained from STM images in Figure 4 (cf. Figure 2 and Figure S7). Considering the equal proportion of (110) and (100) step edges on ion-eroded Pt samples, the STM step density aligns well with the charge in the 0.95 V_RHE_ step oxidation peak. For Pt(111) samples ion-eroded at 600 K or higher, a good match is also found with the charge in the 1.05 V_RHE_ step oxidation peak. For samples prepared at lower temperatures, the step oxidation charge seems to diverge beyond 4 e^–^ per step site. This effect cannot be reasonably assigned to step oxidation and should be interpreted as an onset of terrace oxidation. Indeed, experiments in a sulfate-free electrolyte report the onset of terrace oxidation via a reversible place-exchange process in the form of a sharp peak at a potential of less than 100 mV higher than the potential of (110) step oxidation.^46,55^ Such small separation of step and terrace oxidation peaks cannot be resolved in experiments with high sulfate concentrations.^53^

**Figure 4:**
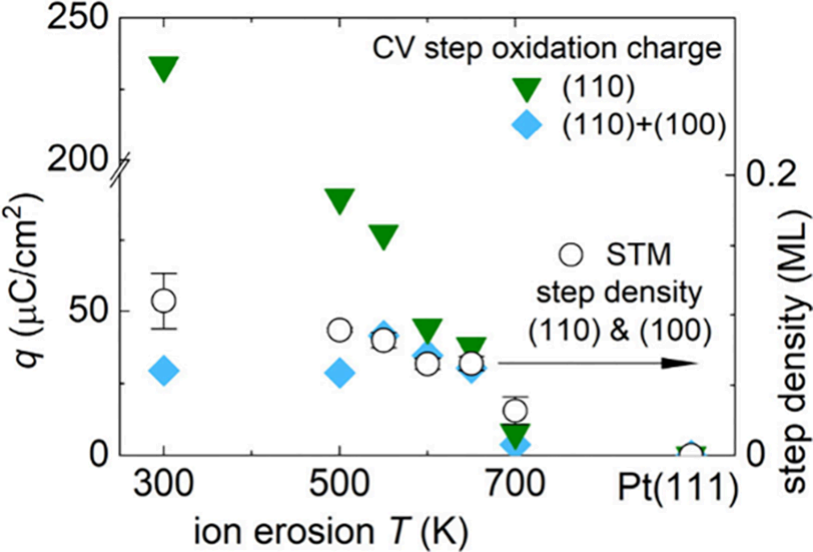
Structure–property relationships in surface oxidation. Left axis (full symbols): charge in the step oxidation peaks in the linear sweep voltammograms of the ion-eroded Pt(111) samples. Right axis (open symbols): (110) and (100) step densities as determined from STM images. The (110) and (100) step densities are equal. Scales of the step oxidation charge (left axis) and step density (right axis) are linked assuming a charge of 2 e^–^ per step site. Note the axis break on the left axis.

We propose that the observed low-potential onset of terrace oxidation can be related to the distinct morphology of the ion-eroded Pt(111) samples, particularly to surface corrugation. In the STM images in Figure 1 and Figure S1, we observe a network of ridges separating the erosion pits. At the points of ridge intersection, monolayer- or multilayer-high Pt islands form. The density of such islands increases with decreasing the erosion temperature, until, at 300 K, Pt islands on ion-eroded samples dominate the surface morphology. Monolayer-high Pt islands feature small lateral sizes (<5 nm), which can be unfavorable for sulfate adsorption (cf. refs 40 and 56 ). Alternatively, a low-potential oxidation channel can be provided due to a significant fraction of step edge atoms in such islands. Eventual contribution of kink sites to the low-potential oxidation of terraces seems to be less relevant, because the relative kink concentration on ion-eroded Pt(111) samples is constant regardless of the ion-erosion temperature (cf. Figure S7e).

In the context of the above discussion, Pt(111) ion-eroded at 300 K [room temperature (RT)] exhibits certain differences from the samples ion-eroded at elevated temperatures. In the blank CV, both (110) and (100) step peaks exhibit broadening, and the charge in the (110) peak is a factor of 2 higher than the charge of the (100) peak. These observations may indicate an unstable morphology of the as-prepared samples and morphology relaxation during electrochemical characterization.^51^ Morphology of the sample may also include structural motifs other than steps and kinks, e.g., low-coordinated Pt atoms, single-atom vacancies, and (110) and (100) facets.^40,41,46^ CVs obtained on the Pt(111) ion-eroded at RT resemble the CVs of polycrystalline or poly-oriented Pt(111) samples.^47^ Also, the first LSV scan of Pt(111) ion-eroded at 300 K (Figure 3b) corresponds well to the LSV scans to Pt oxidation potentials reported for poly-oriented or polycrystalline Pt samples.^57^ This indicates that Pt(111) ion-eroded at room temperature can be considered as a model surface for poly-oriented or polycrystalline Pt samples.

Ion-eroded Ru(0001) and Cu(111) have been prepared to illustrate the general applicability of ion erosion for nanopatterning single-crystal electrodes. Ion energy and exposure were kept the same as for the Pt(111) surface, and the substrate temperature was determined to obtain well-developed erosion pits. Blank CVs of singular Ru(0001) and Cu(111) and the CVs of corresponding ion-eroded surfaces are shown in panels a and b of Figure 5. Panels c and d of Figure 5 and larger STM images in Figure S13 show the morphology of ion-eroded samples measured by STM. Ion erosion of Ru(0001) at 700 K yields ∼8% surface steps. Ion erosion does not affect Ru(0001) surface oxidation and reduction peaks at ∼0.2 and ∼0.45 V_RHE_.^58,59^ On the other hand, a cathodic peak at ∼0 V_RHE_ assigned to substitution of adsorbed OH with H^58,59^ is observed to shift to higher potentials (purple arrow in Figure 5a). This illustrates the structural sensitivity of OH/H substitution and its modification in the presence of step edges on Ru(0001).

**Figure 5:**
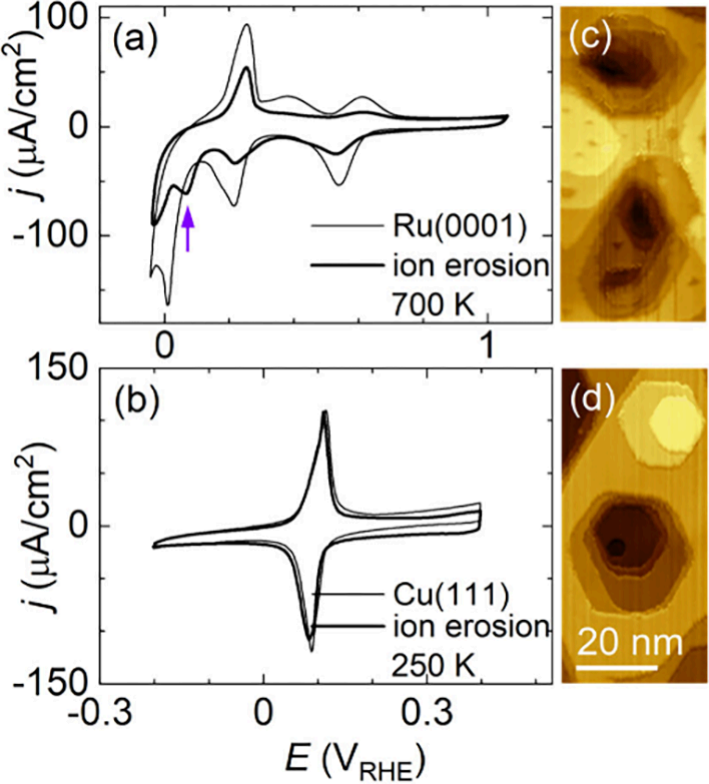
(a) CVs of a singular Ru(0001) (thin line) and Ru(0001) ion-eroded at 700 K (thick line), with 0.1 M HClO_4_, at 50 mV/s. A shifted H_UPD_ peak on the ion-eroded surface is marked by an arrow. (b) CVs of a singular Cu(111) (thin line) and Cu(111) ion-eroded at 250 K (thick line), with 0.1 M KOH, at 50 mV/s. (c and d) STM morphology of ion-eroded Ru(0001) and Cu(111), respectively.

Ion erosion of Cu(111) at 250 K yields ∼4% surface steps, and CVs of singular and ion-eroded surfaces of Cu(111) show small differences. CVs are dominated by OH adsorption/desorption at ∼0.1 V_RHE_.^16^ The charge in the OH adsorption/desorption peak is 88 μC/cm^2^ in both CVs, in good agreement with the value 79 μC/cm^2^ reported in ref 16. On ion-eroded Cu(111), the charge in the double-layer region at ∼0.2 V_RHE_ is higher than on the singular surface. Following the detailed analysis of Cu(111) cyclic voltammetry features from ref 17, we can ascribe this increased charge to a decreased width of Cu(111) terraces on the ion-eroded samples. Other indicators of Cu(111) surface quality, e.g., an absence of a low-potential shoulder in the OH adsorption peak or a negligible intensity of OH adsorption at step or defect sites at ∼0.35 V_RHE_, remain unchanged. This illustrates that ion-eroded Cu(111) surfaces remain well-ordered and clean. An eventual further increase of the step density on ion-eroded Cu(111) can be obtained by increasing the ion-erosion time instead of further decreasing the ion-erosion temperature.^33^

We conclude that ion-eroded single-crystal metal electrodes represent a novel class of model electrodes for electrocatalytic investigations. The morphology of these ion-eroded surfaces is characterized by a specific concentration and density of etch pits, making them qualitatively different from both the morphology of vicinal single crystals and that of single crystals nanostructured through electrochemical methods. Ion-eroded surfaces are, at the same time, rough at a nanometer scale, like nanostructured model electrodes, and constituting solely well-defined adsorption sites, steps and kinks, like vicinal single crystals. The surface roughness and distribution of adsorption sites on ion-eroded surfaces can be precisely controlled by adjusting the sample temperature during ion erosion, and this process is highly reproducible. The unique properties of ion-eroded single-crystal electrodes are illustrated by examples of the Pt(111) surface. For instance, H/OH adsorption and desorption on ion-eroded Pt(111) are significantly influenced by the presence of kinks on the closed step edges inside the etch pits, while surface oxidation is impacted by the finite roughness of the surface. Ion erosion is a versatile technique that can be applied to various metal surfaces, as demonstrated by the successful preparation and electrochemical characterization of ion-eroded Ru(0001) and Cu(111) surfaces. We anticipate that ion-eroded metal single-crystal electrodes will play a crucial role in structure–property investigations of catalytic systems, where the interplay between various types of surface defects becomes important. Future applications of ion erosion in model electrocatalysis may include nanopatterning of (100) or (110) metal surfaces^22^ and nanopatterning of oxides, where preferential sputtering may potentially create new technologically relevant structures.^24,26^ Additionally, ion erosion offers new experimental flexibility as different surface morphologies can be imprinted on one single-crystal substrate. This allows for rapid structure-sensitivity testing, such as comparing the properties of flat, stepped, and disordered surfaces.

## Supplementary Material



## Data Availability

The data that support the findings of this study are presented in the article and the Supporting Information. Source data are provided at Zenodo. DOI: 10.5281/zenodo.15322045.

## Notes

Experimental details, representative STM images of ion-eroded Pt(111) samples, quantification of the erosion pit population, overview of the electrochemical measurement protocol, CVs of ion-eroded Pt(111) samples, charges in the CV of ion-eroded Pt samples, (110) step charge as a function of the (100) step charge, determination of step and kink densities, kink density as a function of the step density, excess reduction charge in a first CV cycle, STM images of as-prepared and emersed samples, LSV scans of ion-eroded Pt(111) samples, step and terrace oxidation charges in the LSVs, and STM images of ion-eroded Ru(0001) and Cu(111) samples (Figures S1–S13), and references (PDF)

## ‡

LEPMI, Université Grenoble Alpes, Université Savoie-Mont-Blanc, CNRS, Grenoble-INP, 38000 Grenoble, France
