# Supplementary material for: Nanopatterning Single-Crystalline Metal Electrodes via Ion Erosion: New Structural Motifs for Model Electrocatalysis
Source: J Phys Chem Lett. 2025 Jul 15;16(29):7420–7. doi: 10.1021/acs.jpclett.5c01465 (PMC12302200; doi:10.1021/acs.jpclett.5c01465)
Supplement: Supplementary file 1 [file jz5c01465_si_001.pdf]

SUPPORTING INFORMATION

Nanopatterning Single-Crystalline Metal Electrodes via Ion Erosion: New Structural Motifs for Model Electrocatalysis

Pankaj Kumar Samal,[1] Jan Škvára,[1] Matyas Výhonský,[1] Lukáš Fusek,[1] Michal Ronovský,[1,+] Viktor Johánek,[1] Maximilian Kastenmeier,[2] Yaroslava Lykhach,[2] Jörg Libuda,[2] Olaf Brummel,[2] Josef Mysliveček[1,*]

[1]*Charles University, Faculty of Mathematics and Physics, Department of Surface and Plasma Science, V Holešovičkách 2, 180 00 Praha 8, Czech Republic*

[2]*Interface Research and Catalysis, ECRC, Friedrich-Alexander-Universität Erlangen-Nürnberg, Egerlandstr. 3, 91058 Erlangen, Germany.*

[+]*Present address: LEPMI, Université Grenoble Alpes, Université Savoie-Mont-Blanc, CNRS, Grenoble-INP, 38000 Grenoble, France*

**josef.myslivecek@mff.cuni.cz*

Experimental Details

Figures S1-S13

References

Experimental Details

Experiments were performed in an UHV surface science apparatus incorporating sample preparation and characterization facilities – ion gun (IQE 11, Specs GmbH), laboratory XPS with dual Mg/Al Kα anode (XRC 1000, Specs GmbH) and a hemispherical analyzer (Phoibos 150, Specs GmbH), LEED (ErLEED 150, Specs GmbH), and a homemade STM operating at RT [1]. Attached to the system is a transfer chamber allowing transfer of the samples prepared and characterized in UHV into an electrochemical cell with stationary electrolyte and back to UHV. Transfer proceeds in an inert atmosphere of 1 atm Ar (Linde, 6.0) [2].

The electrochemical cell was a plastic version (PCTFE, KHP Kunststofftechnik GmbH) of a glass cell used in our previous experiments [3] equipped with a Gamry 2020E potentiostat, a flame-annealed counter electrode (coil of a 1 mm thick Pt wire, Safina, 99.5%) and a leak-less Ag/AgCl reference electrode (eDAQ, type ET 069-1) for acidic electrolytes (H_2SO_4 and $HClO_4$), and a plastic Hg/HgO electrode (ALS, type RE-61AP) for basic electrolytes (KOH). Prior to mounting the EC setup to the load-lock of the UHV chamber, the EC-Cell and bottles for electrolyte were cleaned thoroughly. To do so, the PCTFE cell was soaked overnight in sulfuric acid solution (Merck, EMSURE, 98%) containing NOCHROMIX (Sigma Aldrich) and bottles along with PTFE tubing accessories (ferrules, t-joints, valves) were rinsed with Piranha solution [3:1 mixture of H_2SO_4:H_2O_2 (Sigma Aldrich stabilized for synthesis 30%)]. Afterwards they were washed 3 times with ultrapure water (Merck Millipore®, 18.2 MΩ·cm) and boiled with ultrapure water at ~100°C followed by rinsing with ultrapure water 3 times. This procedure of boiling and rinsing was repeated two more times. After mounting the EC-cell setup, PFA tubing connecting the bottles and the cell was repeatedly rinsed by hot ultrapure water, followed by Ar-purged ultrapure water and, finally, with freshly prepared Ar-purged (30 minutes) electrolyte. Electrolytes were freshly prepared from ultrapure water, H_2SO_4 (Merck Ultrapur®, 96%), $HClO_4$ (Roth Rotipuran® Ultra, 70%), or KOH (pellets, Merck semiconductor grade), and bubbled with Ar for min. 30 min before use. Electrochemical measurement protocols for the samples are illustrated in Figure S3. All reference electrodes (Ag/AgCl and Hg/HgO) were calibrated with respect to reversible hydrogen electrode (RHE), using Pt as working and counter electrodes, purged with high pure hydrogen in respective electrolytes as described by Mukherjee et.al [4]. For measurements on ion-eroded Pt(111) surfaces, potential was further corrected for fluctuations of the leak-less Ag/AgCl reference electrode potential by setting the cathodic (110) step peak to 110 mV_{RHE}. After electrochemical measurement surface of the sample was washed by degassed ultrapure water and was returned to UHV for further characterization.

Investigated electrodes were Pt(111), Ru(0001), and Cu(111) single crystals (MaTecK GmbH) with a nominal diameters of 10 mm (Pt, Cu) and 8 mm (Ru). Ion erosion was performed by Ar^+ ions (Linde, 6.0) on the samples previously cleaned by sputtering and annealing cycles in UHV. For Pt(111) ion erosion was performed by sputtering clean Pt(111) single crystals for 10 min with Ar^+ ions of 1 keV energy 45° off-normal towards (-1-12) direction. Sputtering was performed at Ar background pressure 1.2×10^{-4} Pa. Under these conditions, 6.2 ML of Pt(111) were removed from each prepared sample. Sample temperature during ion erosion was established using radiative heating on the back of the samples (300 – 700 K for Pt, Ru), eventually in combination with LN_2 cooling of the samples (250 K for Cu). Sample temperature was measured by a K-type thermocouple pressed against the sample perimeter. After ion erosion morphology of the samples was measured by STM using electrochemically etched W tips. In some STM images mechanical vibrations become apparent due to a limited mechanical stability of an EC-compatible STM sample holder. When needed for presentation, mechanical vibrations are removed from STM images by means of 2D FFT filtering [5].

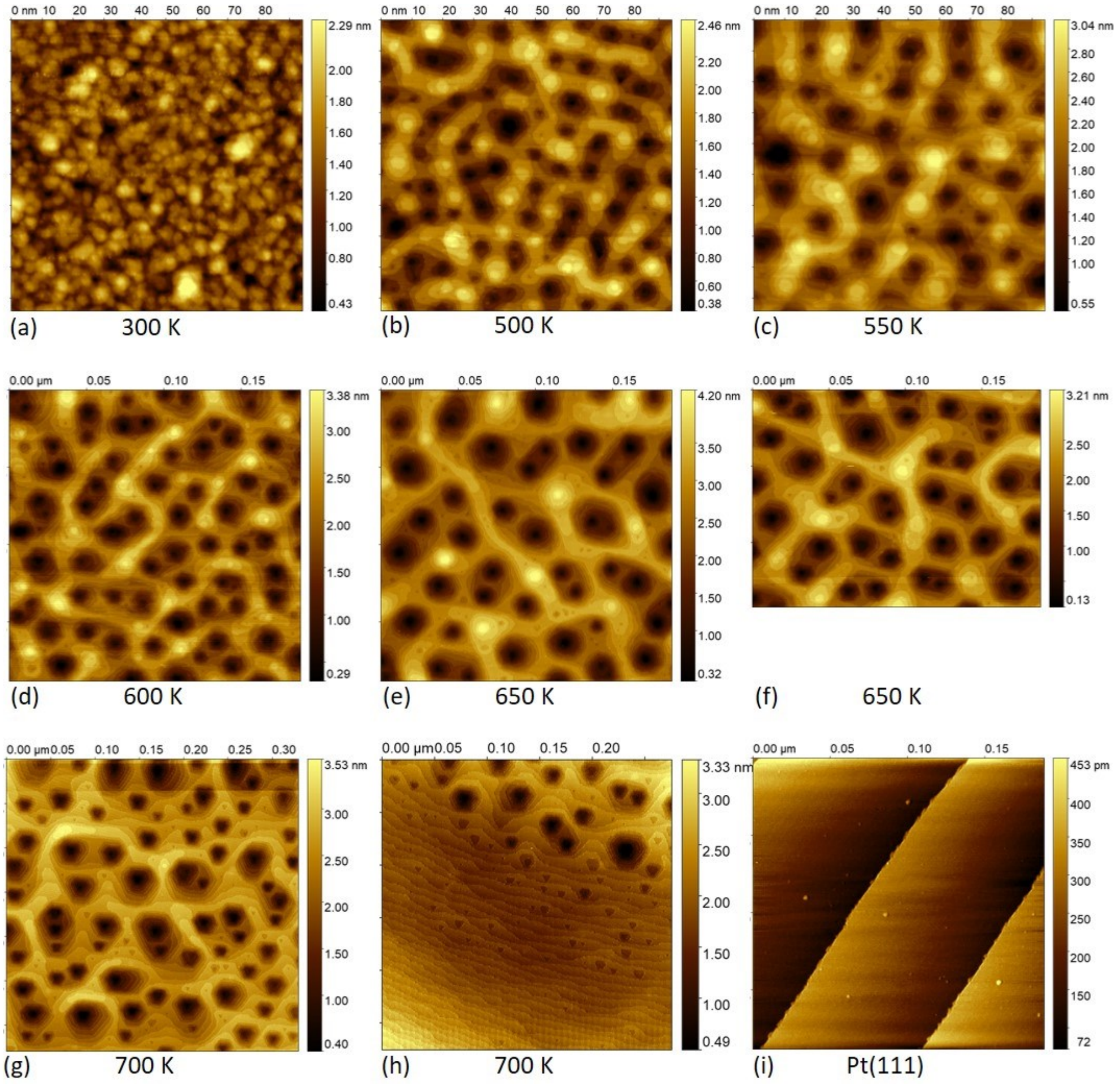

Figure S1: Representative STM images of the as-prepared surfaces of Pt(111) ion-eroded at different temperatures. This selection of images was used for determining the step and kink density of the samples as discussed in the paper (Figures 2, 4, S7, S8). At temperatures 650 K and higher, ion-eroded surfaces are becoming increasingly inhomogeneous.

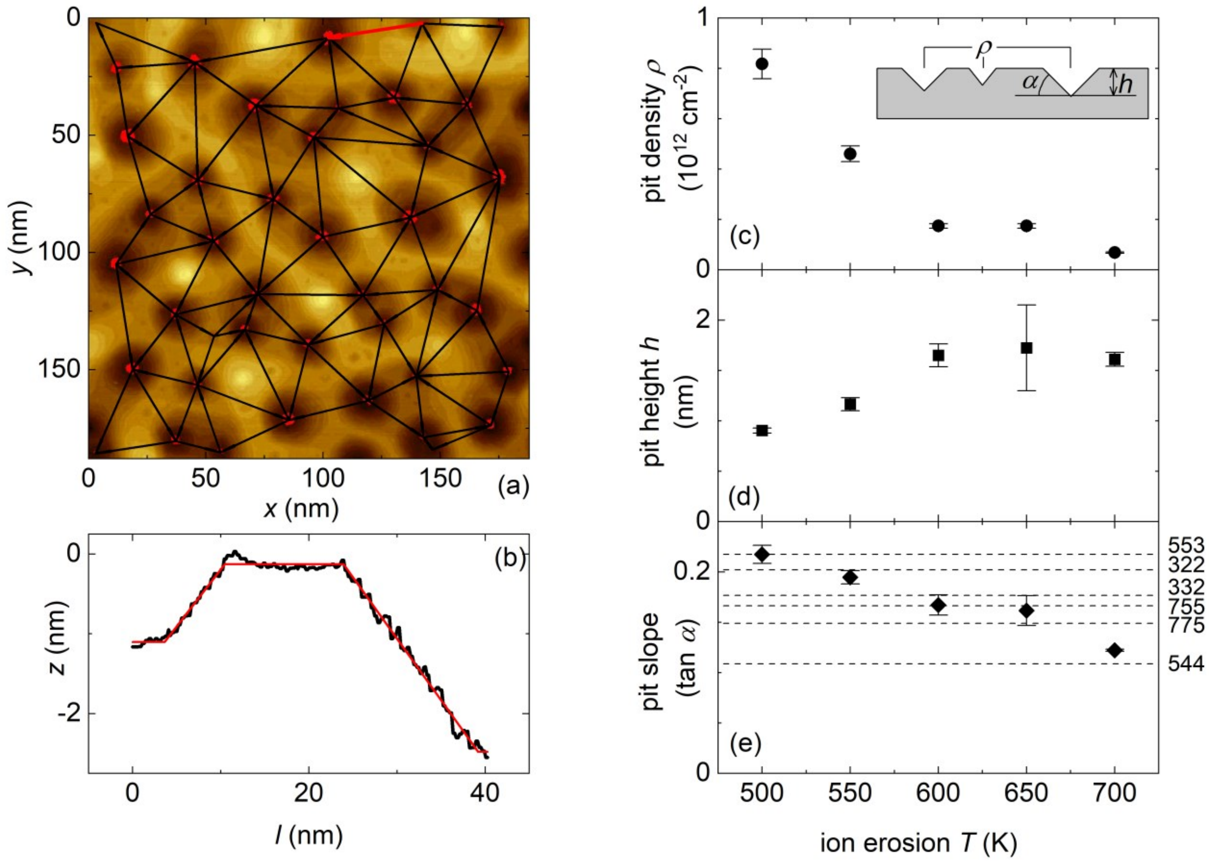

Figure S2: Quantification of the erosion pit population. (a) STM image with pit centers marked by watershed [5] and connected by means of Delaunay triangulation [6]. (b) Sample height profile from STM measured along the edge of a Delaunay triangle (black), and the corresponding linear approximation (least square fit, red [6]). Examples in (a), (b) are given for Pt(111) ion-eroded at 650 K. Average pit density ρ (c), pit height h (d), and pit slope tan α (e) for Pt(111) samples ion-eroded at different temperatures. ρ, h, and α are illustrated in the inset of (c). Error bars in (c)-(e) represent the standard deviations of ρ, h, and tan α distributions obtained from STM images across different, macroscopically distant areas of the samples, and across different samples prepared at the same conditions. Horizontal lines in (e) represent tan α for vicinal Pt(*hkl*) samples of the indicated orientations. Density of the pits is an Arrhenius function of temperature (c) [7]. Characteristic pit height exhibits a maximum at 650 K. At higher temperatures, morphology stabilizing mechanisms start to dominate, and, ultimately, prevent formation of etch pits during ion erosion (d) [7]. Characteristic slope decreases as a function of the increasing ion erosion temperature (e) [7]. Observed slopes correspond to local surface orientations between Pt(553) and Pt(544) (e) [8]. At 650 K and 700 K, ion eroded samples become increasingly inhomogeneous (Figure S1 e-h). At 700 K, areas free of etch pits can be observed (Figure S1 h). This phenomenon is related to the presence of step trains on the Pt(111) surface before ion erosion. When the distance between the pits exceeds the step separation in a step train, formation of the etch pits is suppressed. As a consequence of the observed inhomogeneity, values of h determined at 650 and 700 K are less well-defined.

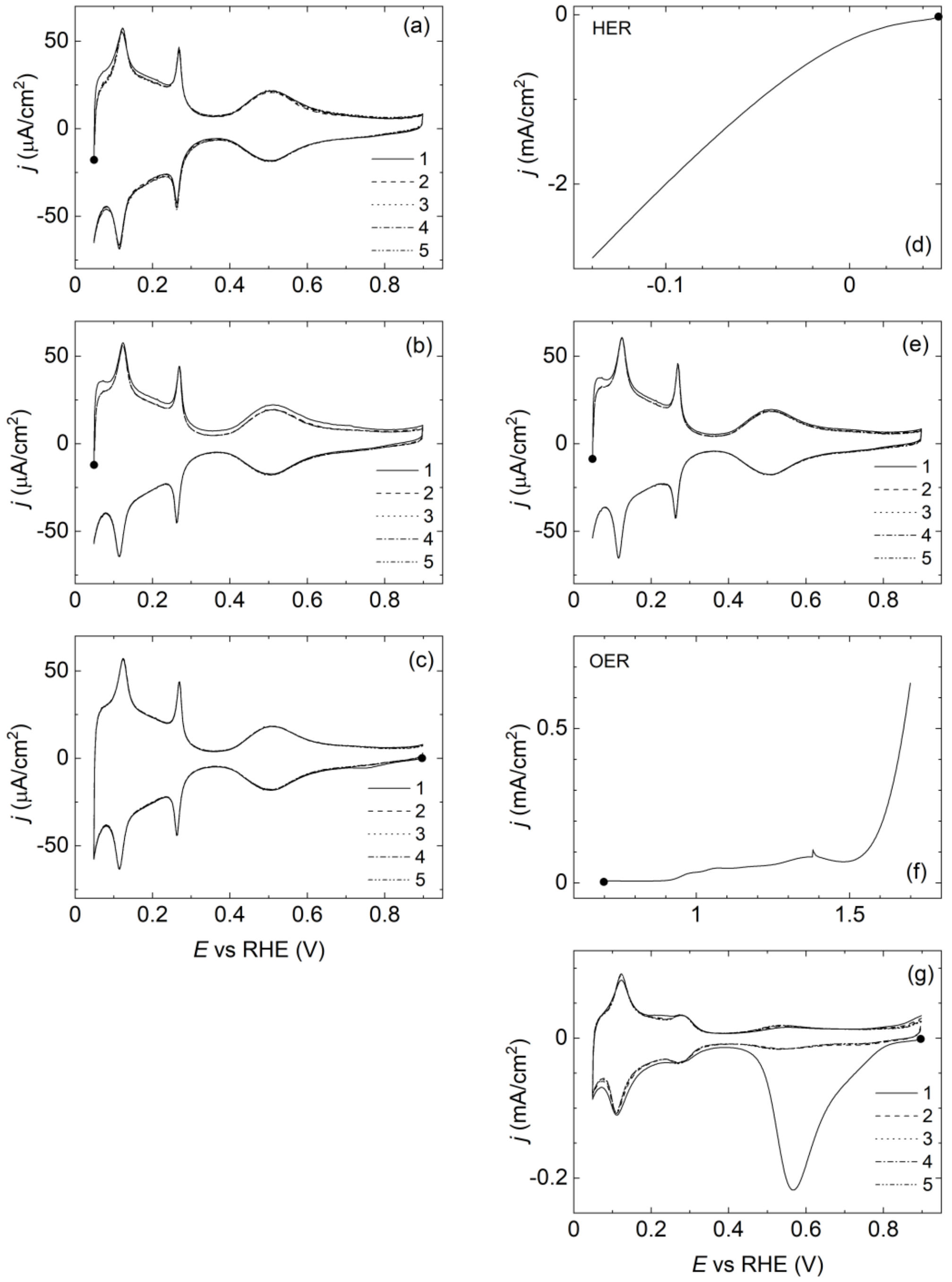

Figure S3: Overview of the electrochemical measurement protocol imposed on the ion-eroded Pt(111) samples in the present study. Example of Pt(111) ion-eroded at 650 K, 0.1 M H_2SO_4, and a sweep rate 50 mV/s. Each sample is subject to electroanalytical treatments (a)-(f). CV measurements in (a)-(c), (e), and (f) consist of 5 cycles each, LSV measurements in (d) and (f) consist of 1 sweep each. Before, and after each treatment the electrochemical contact of the working electrode with the electrolyte is broken and established at a controlled potential (marked by a dot). Between the treatments the electrolyte in the EC cell is exchanged for a fresh one. Presented and evaluated in the paper are CVs from the 5th cycle of (b), and LSVs from (d) and (f).

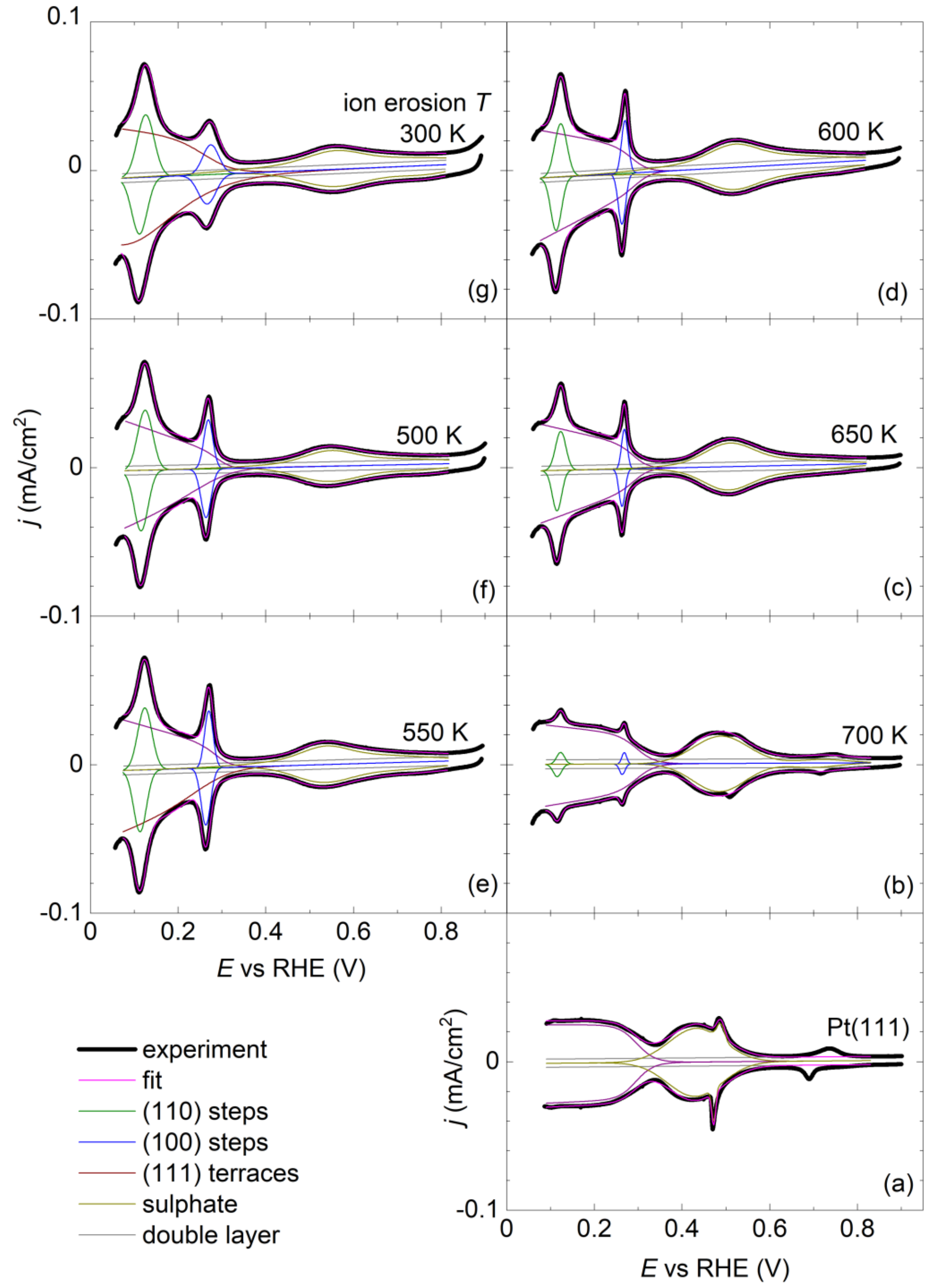

Figure S4: Cyclic voltammograms of the Pt(111) samples ion-eroded at different temperatures (0.1 M H_2SO_4, 50 mV/s). Included is a decomposition to charge contributions assigned to H/OH adsorption and desorption on (111) terraces, (110) and (100) steps, sulfate adsorption and desorption on (111) terraces, and a double layer. Decomposition is obtained by considering the double layer charge of 50 $\mu C/cm^2$, and least-square fitting of 4 Gaussians (1 for each step type, and 2 for the sulfate), and 1 modified step function (for the terrace, [9]) to the experimental data. Total charge in the CVs varies between 200 and 300 $\mu C/cm^2$ which we assign to variations in the wetted area of the samples relative to their geometrical area of 0.785 cm^2 (1 cm diameter).

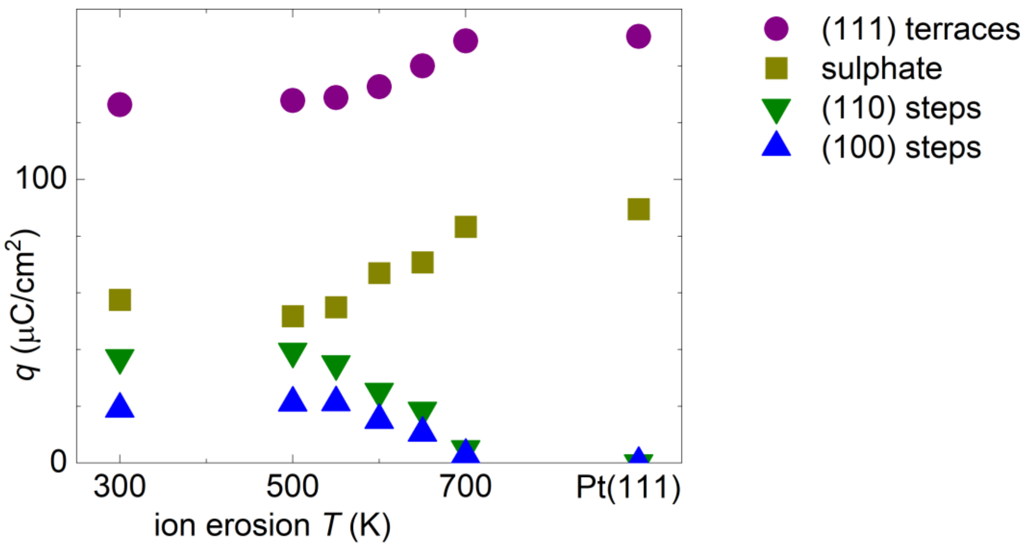

Figure S5: Charges in the CV of ion eroded Pt samples from Fig. S4. Step charges are results of the fits from Figure S4, sulfate charges are obtained as a difference of total charge in CV (integrated numerically between 0.06 and 0.80 V), and (111) terrace charge (integrated numerically between 0.06 and 0.35 V). To compensate for the variations in the wetted area of the samples, charges are normalized to a total charge in CV of 300 μC/cm^2, and a double layer charge of 60 μC/cm^2 [10], [11]. The same normalization applies to charges in Figure 2. All charges are averaged between anodic and cathodic scans.

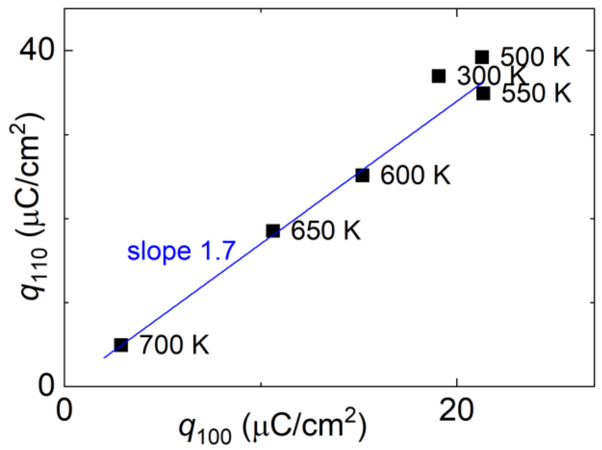

Figure S6: (110) step charge as a function of (100) step charge for Pt(111) samples ion-eroded at the indicated temperatures. Data from Figures 2, S5.

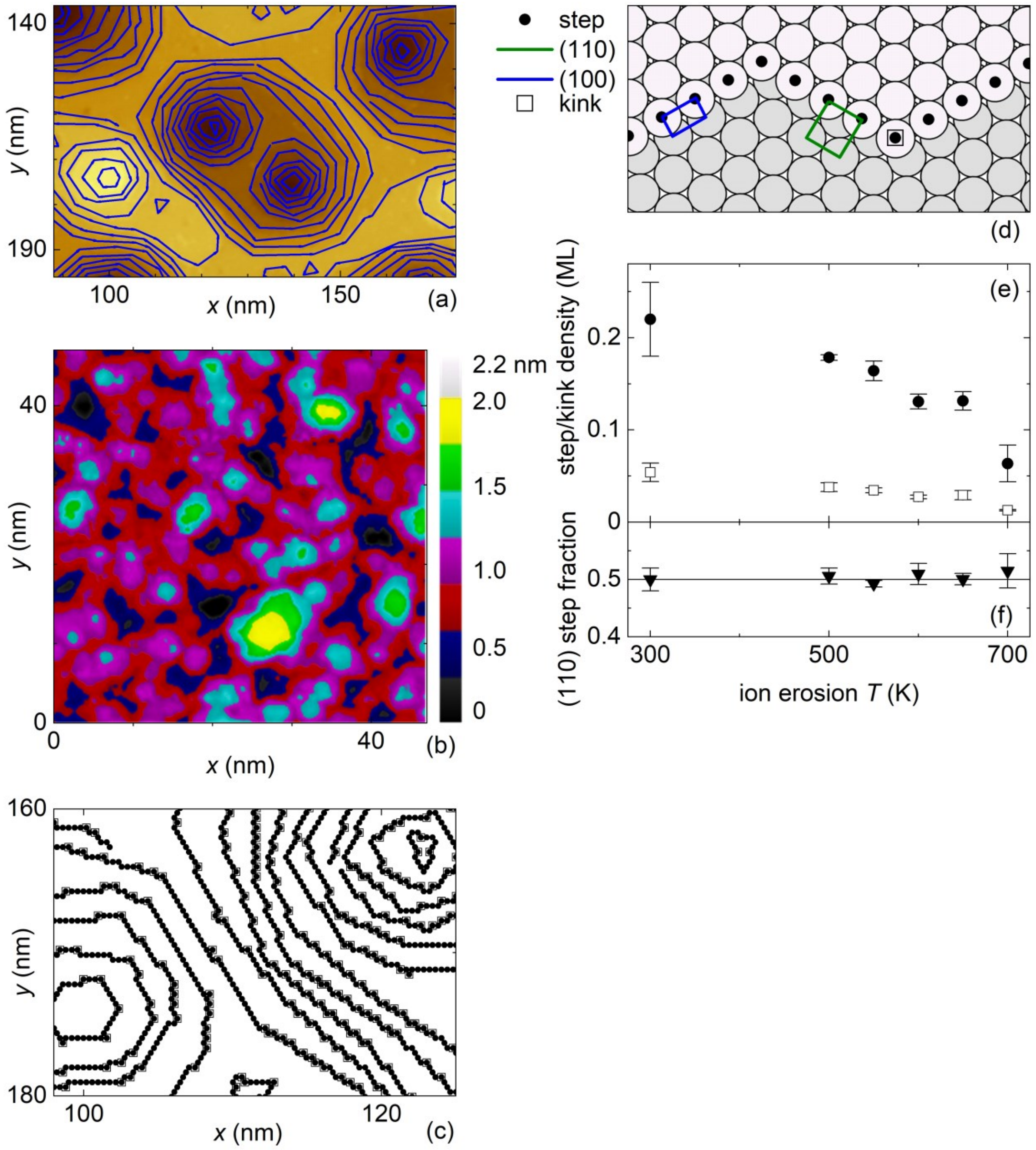

Figure S7: Determination of the step and kink density for the ion-eroded Pt(111) surfaces. For surfaces ion-eroded at *T* between 500 K and 700 K, step edges and kinks are not atomically resolved in the STM images (Figure S1 b-h). Analysis is thus based on an approximation obtained by projection of step edge positions observed in the experiment on an ideal two-dimensional grid of Pt(111) atoms. Outlines of the step edges are marked in the STM images manually using a polyline tool of a vector drawing software [12]. Example in (a) is given for Pt(111) ion-eroded at 650 K, and the manual outline is highlighted in blue.

For the surface ion-eroded at 300 K, even step edges are not resolved in the STM images (Figure S1 a). Positions of the step edges can be estimated by the approach of Ref. [13] where the step positions are modeled by contours of constant height superimposed onto the STM image. Example for the sample ion-eroded at 300 K is given in (b). Height contours are separated by the step height on Pt(111), i.e. 0.277 nm [5]. Once modeled, contours are outlined manually as in (a).

Manual outlines saved from the vector drawing software (.svg file format) are further processed using a home-written computer script. Outlines are decomposed into linear segments and projected on a properly oriented and scaled hexagonal grid corresponding to positions of Pt atoms on a Pt(111) plane. Grid nodes in the closest vicinity of the outlines are marked as step atoms. Example in (c) is given for the outlines from (a). Top view of a step edge model with step atoms marked by black dots is given in (d).

Step density (e) is evaluated as a density of step atoms relative to density of Pt(111) atoms (1.50×10^{15} cm^{-2}). Mutual positions of the step atoms are further analyzed to determine the density of kinks, and the fraction of (110) step segments. A kink is identified as a step atom having two step atom neighbors at a mutual angle of 120°, and pointing downhill (d, black square). Downhill direction is identified based on the local height measured in a smoothed STM image (Gaussian blur, [5]). Kink density (e) is evaluated as a density of kinks relative to density of Pt(111) atoms. (110) and (100) step segments are identified as step atoms having a step atom neighbor at azimuthal angles (0, 120, 240)° and (60, 180, 300)°, respectively (d). (110) step fraction (f) is determined as a number of (110) step segments relative to a sum of (110) and (100) step segments. (110) step orientation is determined based on STM image of Pt(111) ion-eroded at the highest temperature (700 K, Figure S1 g) where some prevalence of one step orientation, which we assume to be (110), becomes apparent. Lateral shifts between Pt(111) terraces of different heights are neglected. Application of the script results in a small fraction of mis-assigned step atoms, kinks, and step segments which we estimate to <5% of each of the evaluated quantities in (e, f).

Data points in (e, f) are obtained from corresponding STM images in figure S1 a, b, c, d (300 K, 500 K, 550 K, 600 K), or as average values from Figures S1 e, f (650 K), and S1 g, h (700 K). For Pt(111) ion eroded at 700 K, obtained STM images do not allow to fully characterize the inhomogeneous nature of the surface. Coverage of step trains (Figure S1 h) is thus considered 50%, and (110) step fraction in the step trains is considered 0.5 (all step train orientations). Error bars in (e), (f) represent a standard deviation due to a subjective error in manual outlining, and due to inhomogeneity of the samples. For the 300 K sample, error bars in (e) are set to 20 % to account for the simplifications taken in modeling the step edge positions (b).

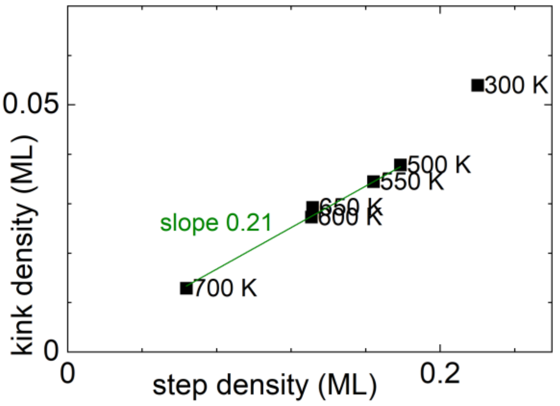

Figure S8: Kink density as a function of step density for Pt(111) samples ion-eroded at the indicated temperatures. Data from Figure S7 e.

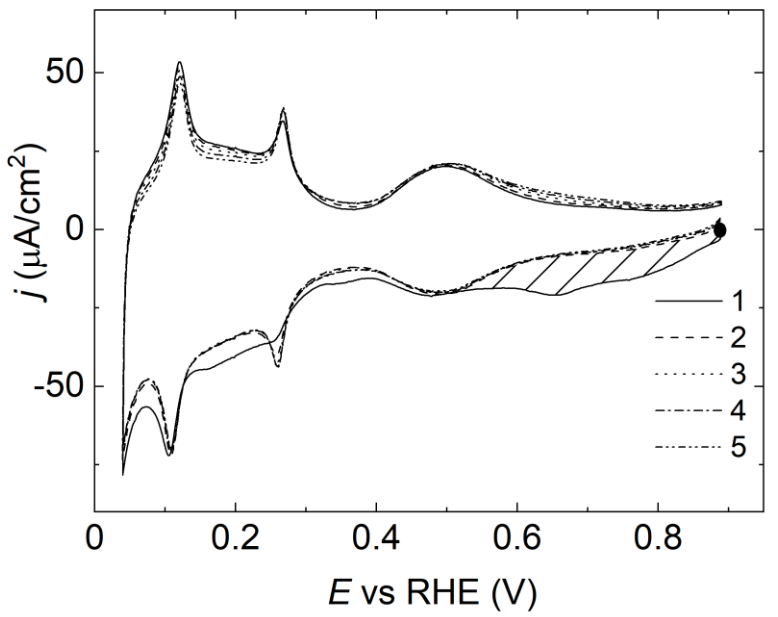

Figure S9: Excess reduction charge in the first CV cycle upon a sample transfer from UHV (0.1 M H_2SO_4, 50 mV/s). Charge is measured relative to the charge in subsequent CV cycles (hatched area, 60 μC/cm^2, [14]). This is a separate experiment dedicated to determining the excess reduction charge, different from the measurement protocol for other samples (first contact potential at 0.9 V, compared to 0.05 V in Figure S3).

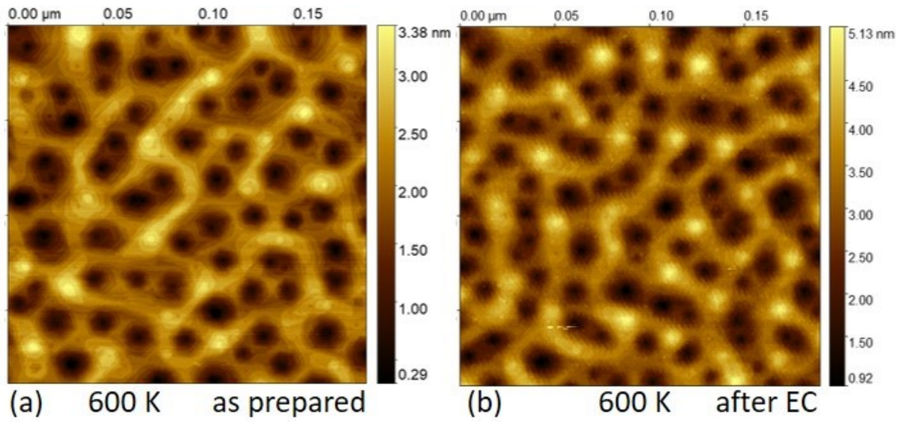

Figure S10: (a) STM image of an as-prepared Pt(111) sample ion-eroded at 600 K. (b) STM image of (a different) Pt(111) sample ion-eroded at 600 K and emersed upon CV measurement (Figures S3 a-c).

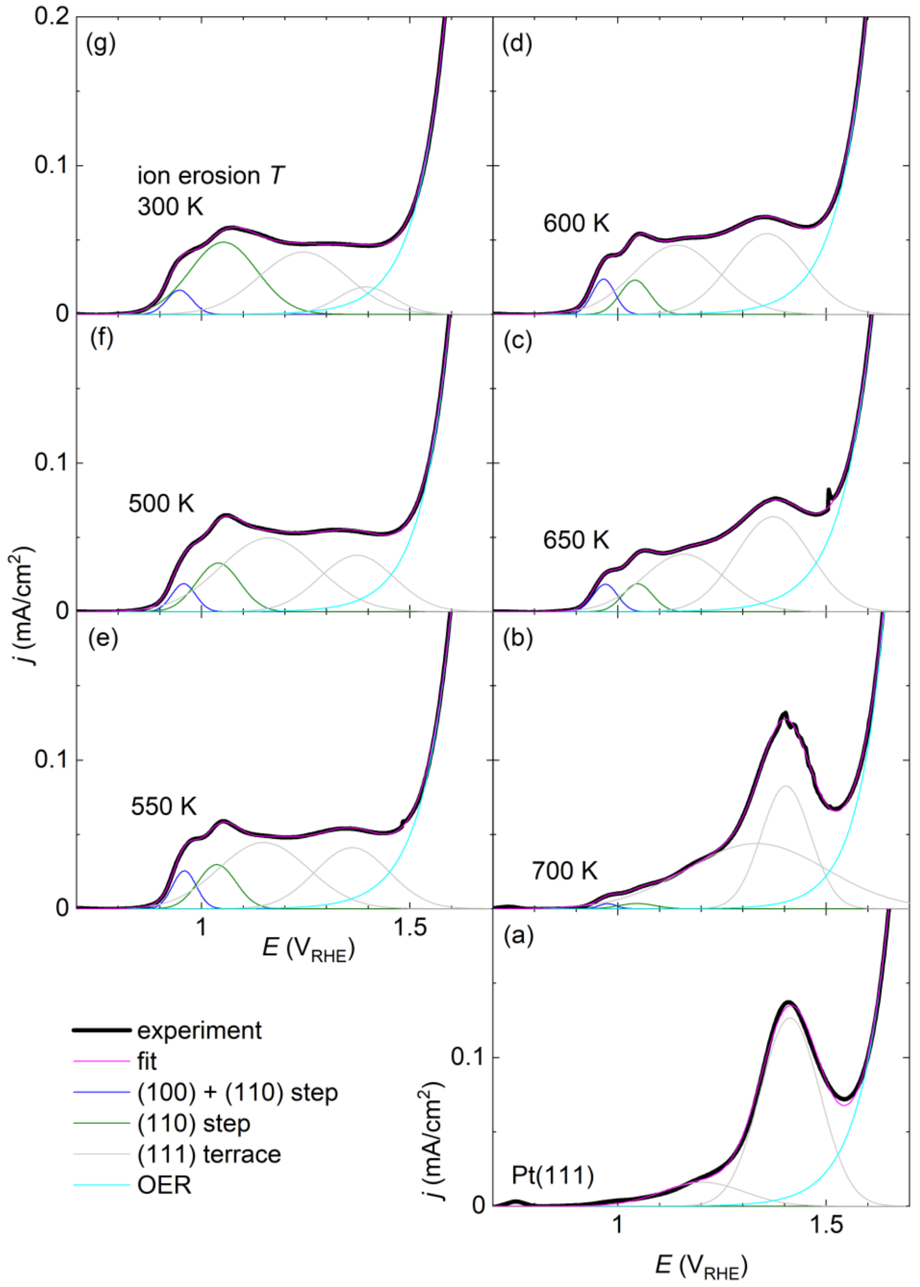

Figure S11: LSV scans of the Pt(111) samples ion-eroded at different temperatures (0.1 M H_2SO_4, 50 mV/s). Included is a decomposition to charge contributions assigned to oxidation of surface steps and terraces, and to OER. Decomposition is obtained by least-square fitting of 4 Gaussians (2 for steps and 2 for the terrace), and an exponential for the OER. Range of the fitted data is limited to 350 μA/cm^2. For Pt (111) sample, only 2 Gaussians are fitted. For all samples, fits are obtained without restrictions imposed on peak position or width. LSV measurements at a high sulfate concentrations do not provide a well-resolved low-potential terrace oxidation peak (at about 1.1 V_{RHE}, see text and Ref. [15]), and attempts to include a fifth peak in the fitting fail. Total oxidation charge in the LSVs varies between 500 and 600 μC/cm^2 which we assign to variations in the wetted area of the samples relative to their geometrical area of 0.785 cm^2 (1 cm diameter).

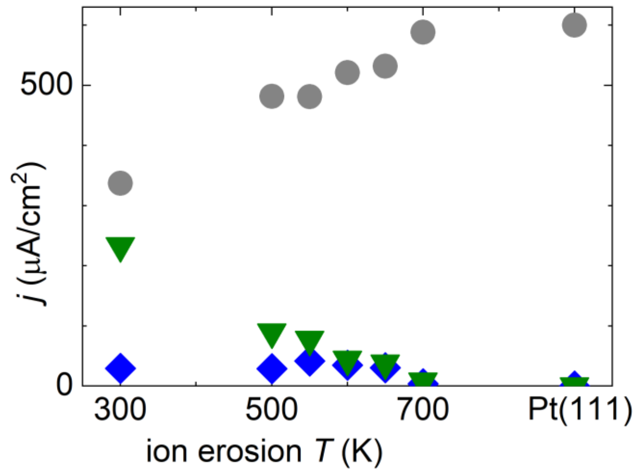

Figure S12: Step and terrace oxidation charges in the LSVs of ion eroded Pt samples from Fig. S11. Step charges are results of the fits from Figure S11, terrace charges are obtained as a difference of total oxidation charge in LSVs (integrated numerically between 0.8 and 1.6 V, and excluding double layer and OER charges), and step charges. To compensate for the variations in the wetted area of the samples, charges are normalized to a total oxidation charge in LSV of 600 μC/cm^2 [15]. The same normalization applies to charges in Figure 4.

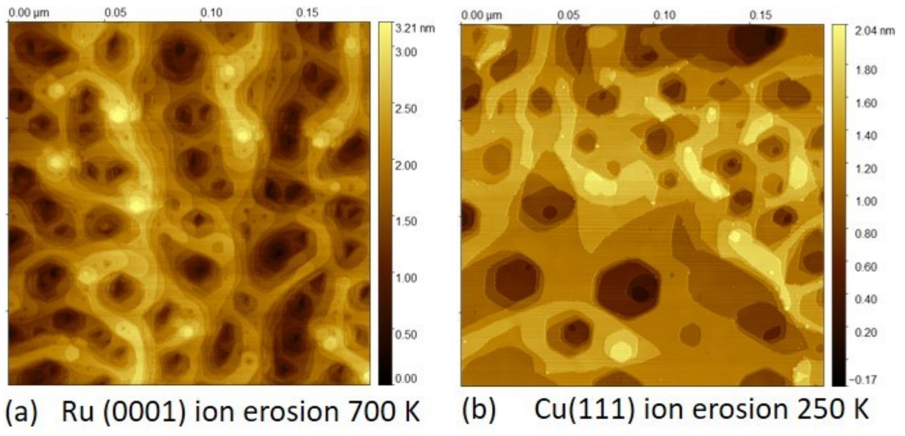

Figure S13: (a) STM image of a Ru(0001) sample ion eroded at 700 K. (b) STM image of a Cu(111) sample ion-eroded at 250 K.

References

[1] V. Stetsovych, F. Pagliuca, F. Dvořák, T. Duchoň, M. Vorokhta, M. Aulická, J. Lachnitt, S. Schernich, I. Matolínová, K. Veltruská, T. Skála, D. Mazur, J. Mysliveček, J. Libuda, and V. Matolín, "Epitaxial Cubic Ce_2O_3 Films via Ce–CeO_2 Interfacial Reaction," *J. Phys. Chem. Lett.* 4, 866, 2013, doi:10.1021/jz400187j.

[2] F. Faisal, C. Stumm, M. Bertram, F. Waidhas, Y. Lykhach, S. Cherevko, F. Xiang, M. Ammon, M. Vorokhta, B. Šmíd, T. Skála, N. Tsud, A. Neitzel, K. Beranová, K. C. Prince, S. Geiger, O. Kasian, T. Wähler, R. Schuster, M. A. Schneider, V. Matolín, K. J. J. Mayrhofer, O. Brummel, and J. Libuda, "Electrifying model catalysts for understanding electrocatalytic reactions in liquid electrolytes," *Nat. Mater.* 17, 592, 2018, doi:10.1038/s41563-018-0088-3.

[3] A. Simanenko, P. K. Samal, R. Hübsch, J. Škvára, J. Yang, M. Kastenmeier, F. Winkler, T. Skála, N. Tsud, S. Mehl, J. Mysliveček, O. Brummel, Y. Lykhach, and J. Libuda, "Origin of the Low Overpotential for Isopropanol Oxidation on Pt–Ru Electrocatalysts," *ACS Energy Lett.* 9, 4875, 2024, doi:10.1021/acsenergylett.4c01987.

[4] D. Mukherjee, P. M. Austeria, and S. Sampath, "Two-Dimensional, Few-Layer Phosphochalcogenide, $FePS_3$: A New Catalyst for Electrochemical Hydrogen Evolution over Wide pH Range," *ACS Energy Lett.* 1, 367, 2016, doi:10.1021/acsenergylett.6b00184.

[5] D. Nečas and P. Klapetek, "Gwyddion: an open-source software for SPM data analysis," *Open Phys.* 10, 181, 2012, doi:10.2478/s11534-011-0096-2.

[6] P. Virtanen, et al., "SciPy 1.0: fundamental algorithms for scientific computing in Python," *Nat. Methods* 17, 261, 2020, doi:10.1038/s41592-019-0686-2.

[7] M. Kalff, G. Comsa, and T. Michely, "Temperature dependent morphological evolution of Pt(111) by ion erosion: destabilization, phase coexistence and coarsening," *Surf. Sci.* 486, 103, 2001, doi:10.1016/S0039-6028(01)01015-9.

[8] V. Climent and J. Feliu, "Single Crystal Electrochemistry as an In Situ Analytical Characterization Tool," *Annu. Rev. Anal. Chem.* 13, 201, 2020, doi:10.1146/annurev-anchem-061318-115541.

[9] I. T. McCrum and M. J. Janik, "Deconvoluting Cyclic Voltammograms To Accurately Calculate Pt Electrochemically Active Surface Area," *J. Phys. Chem. C* 121, 6237, 2017, doi:10.1021/acs.jpcc.7b01617.

[10] A. Rodes, K. El Achi, M. A. Zamakhchari, and J. Clavilier, "Hydrogen probing of step and terrace sites on Pt(S)-[n(111) × (100)]," *J. Electroanal. Chem. Interfacial Electrochem.* 284, 245, 1990, doi:10.1016/0022-0728(90)87077-W.

[11] J. Clavilier, K. El Achi, and A. Rodes, "In Situ Probing of Step and Terrace Sites on Pt(S)-[n(111)×(111)] Electrodes," *Chem. Phys.* 141, 1, 1990, doi:10.1016/0301-0104(90)80014-O

[12] "Inkscape." [Online]. Available: https://inkscape.org/.

[13] L. Jacobse, M. J. Rost, and M. T. M. Koper, "Atomic-Scale Identification of the Electrochemical Roughening of Platinum," *ACS Cent. Sci.* 5, 1920, 2019, doi:10.1021/acscentsci.9b00782.

[14] A. Rodes and J. Clavilier, "Electrochemical study of step reconstruction on platinum surfaces belonging to the [01] zone between Pt(311) and Pt(111)," *J. Electroanal. Chem.* 344, 269, 1993, doi:10.1016/0022-0728(93)80060-U.

[15] A. Björling, E. Herrero, and J. M. Feliu, "Electrochemical Oxidation of Pt(111) Vicinal Surfaces: Effects of Surface Structure and Specific Anion Adsorption," *J. Phys. Chem. C* 115, 15509, 2011, doi:10.1021/jp204306k.
